# Infanticide in Atlanta

**Published:** 1884-09

**Authors:** 


### INFANTICIDE IN ATLANTA.

Abandonment of illegitimate offspring is murder by piecemeal, and should be punished accordingly by law.

As an appropriate sequel to our comments on criminal abortion in the last number of THE JOURNAL, it devolves upon us to notice two recent cases of abandonment of the fruits of illicit intercourse which have been made public in this city. There are indications of a more humane disposition on the part of mothers in trying to conceal their disgrace by turning over the responsibility of caring for the children to others; yet the utter insensibility to every sentiment which should control a woman in watching over and providing for her offspring is at least strikingly demonstrated by one being left with a negro family in a dirty hovel,and by the other being abandoned on the street, to be picked up and carried to the Benevolent Home. The fate of that consigned to negro guardians is recorded in the coroner's verdict of neglect and starvation, and what is to become of the other infant bereft of maternal care may be readily foreseen, unless measures are promptly taken by the authorities in the case, as we learn have been adopted, in the fatal result, to ferret out the guilty parties and give them the alternative of marriage to avoid the prosecution by law. In this event the child may be restored to its parents, to receive the attention requisite for its preservation.

We are aware that some people are found as apologists of the culprits in concealing the parentage of their offspring. But one offense cannot excuse another, and if the gratification of improper desires is followed by conception and the birth of a child, it is the part of true womanhood to abide by the consequences. In the rejection of her child, a woman, whether married or single, unsexes herself, and is reduced in the scale of creation below the grade of brutes, as even the most debased animals manifest the instinct of love for their offspring, and it is well known that the most timid specimens of quadrupeds and birds will resist encroachments upon the hiding place of their young.

Thus it turns out that the elegant and refined female of the human race, whose passions overleap the bounds of propriety, or who is misled by the approaches of a seducer, falls so far beneath the types of creation and of conformity to nature's requirements, as to go any lengths that may be made to subserve the concealment of her illicit intercourse.

Shame upon the woman who indulges her propensities, with the intention to sacrifice the fruits of her temporary gratification, either by the destruction of the unborn child. or by consigning it upon delivery to those who will slowly but certainly prove as helpmeets for its accompaniment to the grave. This issue must be met fairly and squarely.

Is not such abandonment of children by their parents fairly included under the head of murder, it being done clearly with intent to kill, as under the law of probabilities no other result than the death of the child under such circumstances can be anticipated? Are not those entrusted with the faithful execution of our laws bound to set detectives on the track of every mother and of every father who willfully and knowingly leave their child to waste away by the slow but sure process of neglect? Every honest young woman in the land becomes implicated by the doubt as to the source of a foundling, and every respectable family should be interested in exposing these unnatural and inhuman acts. The record of forty-five dead bodies of children, ranging in age from a few hours up to two months, found in the city of New York within four and a half months, does not call more loudly for action than does the cruel intention of murder on the part of mothers who discard their offspring, and let them be found among the high or the low class of the community, they should be brought to justice. The punishment of the guilty is demanded for the protection of the innocent.

The history of baby-farming in Northern cities shows that those institutions which guarantee the largest mortality are the best patronized; and it is fair to infer that those mothers amongst us who wish to be rid of their children select keepers who may be relied upon to treat them with masterly inactivity. They are murderers, and should be so treated.

